# Orthodontic Management of a Growing Patient With a Skeletal Class III Malocclusion and an Anterior Open Bite: A Case Report

**DOI:** 10.7759/cureus.70731

**Published:** 2024-10-02

**Authors:** Sana Bint Aziz, Garima Arora, Gurkeerat Singh, Puneet Batra, Ashish K Singh

**Affiliations:** 1 Orthodontics and Dentofacial Orthopaedics, Manav Rachna Dental College, Faridabad, IND; 2 Orthodontics and Dentofacial Orthopaedics, Sudha Rustagi College of Dental Sciences and Research, Faridabad, IND

**Keywords:** chin cup, facemask therapy, hyrax expander, open bite, rapid maxillary expansion, skeletal class iii

## Abstract

Class III malocclusion presents many challenges due to its varying elements of imbalance in skeletal, dental, and soft tissues. This necessitates a comprehensive treatment plan, including growth modification during the pre-pubertal growth phase, and long-term retention to reduce the chances of orthognathic surgery later. The components of Class III malocclusion include maxillary retrognathism, mandibular prognathism, or, in some cases, a combination of both. This article presents a case report of a pre-pubertal female patient, 11.5 years old, with skeletal Class III malocclusion, who sought orthodontic treatment for a backwardly positioned upper jaw. The case was complicated due to a vertical growth pattern with an FMA of 36 degrees, a constricted maxilla in a retrognathic position, unerupted maxillary canines, mandibular anterior crowding, and an anterior open bite. During the first phase of treatment, maxillary protraction with rapid palatal expansion was performed using a facemask appliance and a bonded Hyrax expander. Corrective orthodontics with a fully fixed labial appliance followed after the retention period for maxillary expansion. A functionally stable occlusion was achieved with good vertical control. The patient was satisfied with an aesthetically pleasing smile at the end of the treatment. However, orthognathic surgery might be needed to address the problem of a prominent chin and severe vertical growth pattern. Furthermore, the patient was recommended to wear a vertical pull chin cup for 12 hours daily to prevent any further mandibular growth. Early intervention yielded favorable outcomes, including improvement in skeletal discrepancy, spontaneous eruption of maxillary canines, and an aesthetically enhanced facial profile. The present case report highlights the importance of timely management of malocclusion in growing patients.

## Introduction

Patients with Class III malocclusion may present either with a prognathic mandible, a retrognathic maxilla, or a combination of both [1]. Apart from the skeletal discrepancy, Class III malocclusion may also result from dental inconsistencies, such as an anterior crossbite. The orthodontic treatment for skeletal Class III patients is quite challenging, as the problem worsens with time. Early treatment in growing patients may provide skeletal correction with the use of orthopedic appliances; however, camouflage treatment or orthognathic surgery is required in adult patients, depending on the severity of the malocclusion [2].

Class III malocclusion is often linked with genetic as well as environment-related factors and is more prevalent in Asians, with an incidence rate of 13%, compared to Europeans, who have an incidence rate of 1% to 5% [3]. Maxillary retrognathism is found in almost 45.5% of Class III malocclusion cases [4]. The most effective way to treat a retrognathic maxilla in pre-pubertal patients is protraction facemask therapy, along with rapid maxillary expansion, as most Class III cases often present with maxillary constriction as well [5]. Early facemask therapy results in the correction of the occlusal discrepancy, skeletal protraction of the maxilla by 1-2 mm, and forward displacement of maxillary anterior teeth, along with lingual tipping of the mandibular anterior teeth [1]. It is important to follow up with the patient until their growth is complete due to the high likelihood of relapse after treatment. A previous study [6] found that early treatment of Class III malocclusion with a facemask, rapid maxillary expansion appliance, and posterior bite planes resulted in long-term, stable, and aesthetically pleasing outcomes, reducing the need for future surgery. Adult patients with a constricted maxilla may require surgically-assisted rapid maxillary expansion (SARPE). Recently, the development of mini-screw-assisted rapid palatal expansion (MARPE) has eliminated the need for surgery in adult patients for expansion [7].

It is a known fact that Class III malocclusion develops early in life and can be identified during the primary or mixed dentition stage. Therefore, early intervention during the pre-pubertal growth phase is recommended for favorable growth modifications [2]. The present case report describes early treatment with a protraction facemask and rapid maxillary expansion in a pre-pubertal female patient with unerupted maxillary canines and an open bite tendency.

## Case presentation

Diagnosis

**Figure 1:**
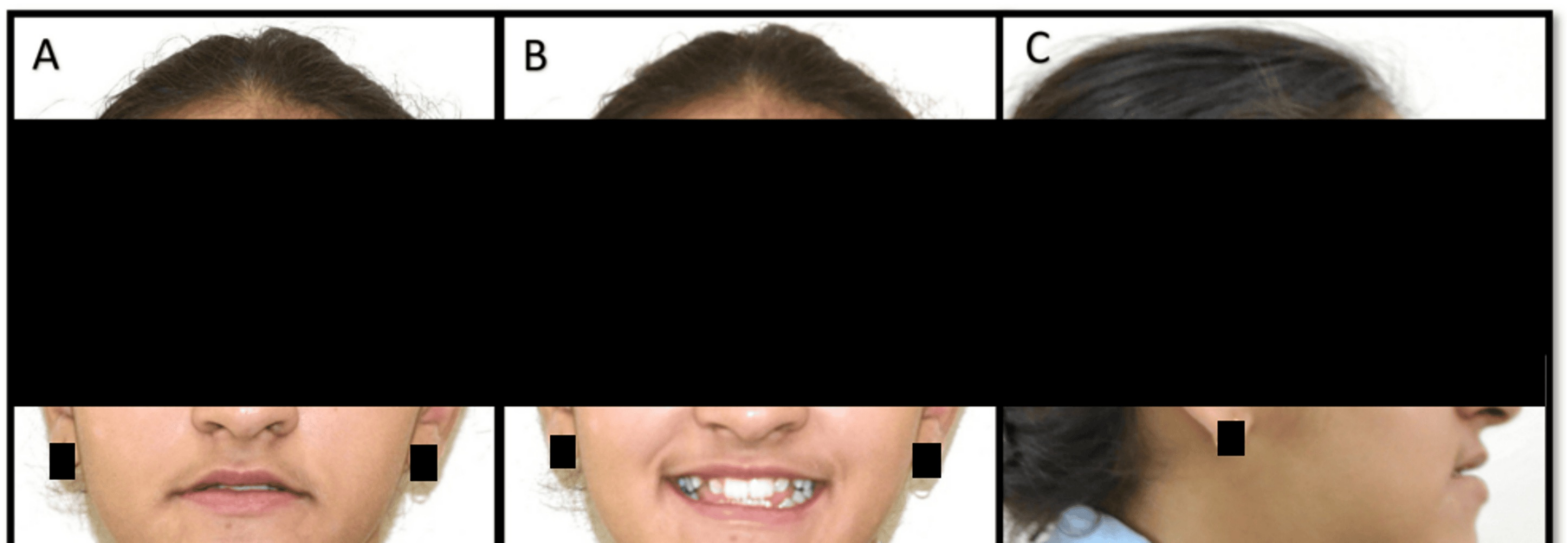
Pre-treatment extra-oral photographs A) Frontal at rest; B) Frontal smiling; C) Lateral at rest

**Figure 2:**
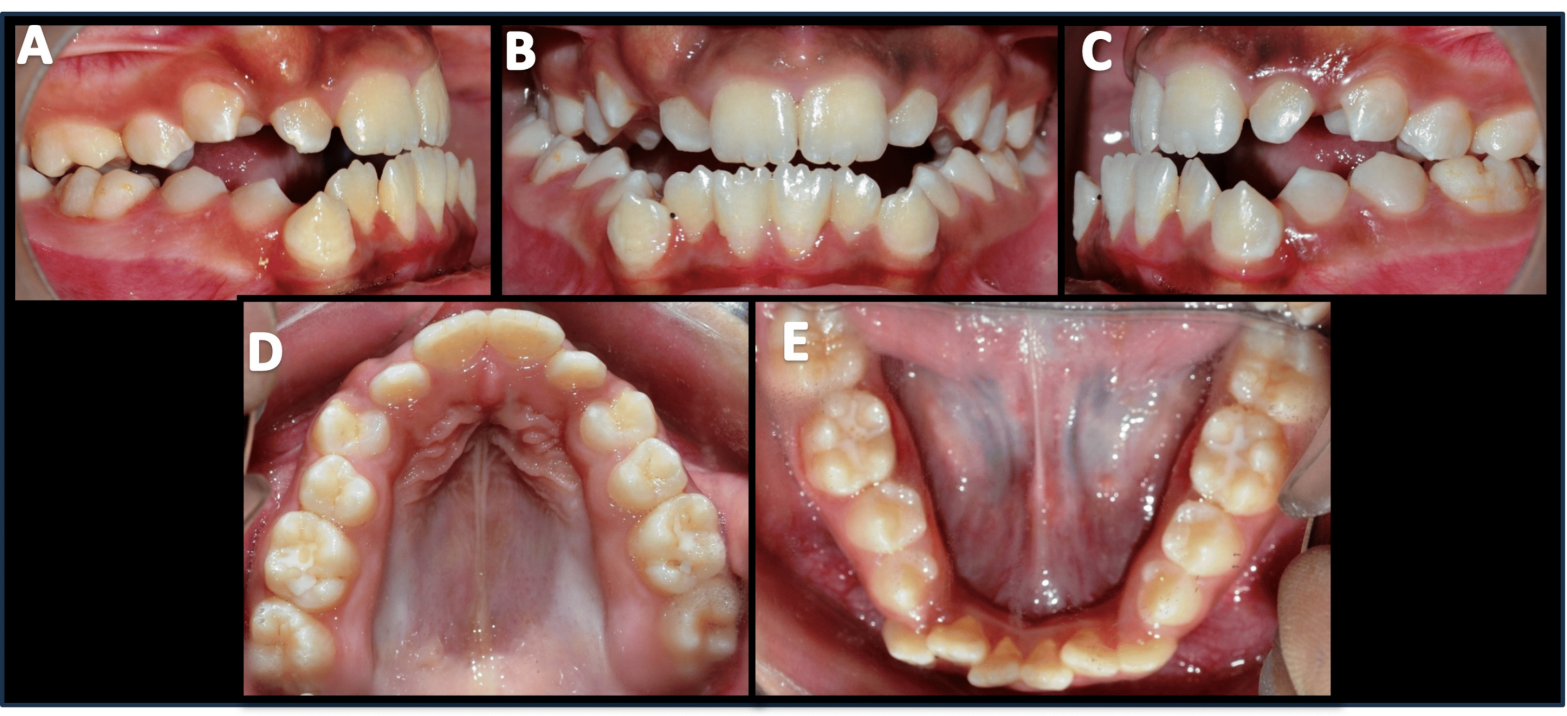
Pre-treatment intra-oral photographs A) Right buccal view; B) Frontal view; C) Left buccal view; D) Maxillary occlusal view; E) Mandibular occlusal view

A prepubertal female patient, aged 11.5 years, reported to the Outpatient Department of Orthodontics and Dentofacial Orthopaedics at our institute with the chief complaint of a forwardly placed lower jaw. She presented with a straight profile, anterior facial divergence, and a shallow mento-labial sulcus. She had an average nasolabial angle, and the mandibular plane angle was high. The poor development of the mid-face region indicated a maxillary deficiency (Figure 1). On intraoral examination, the patient presented with dentoalveolar Angle’s Class I, Dewey’s type 3 malocclusion. She had a constricted maxillary arch with unerupted maxillary canines and labial bulges present on both sides. There was severe crowding in the mandibular arch, with distally tipped mandibular canines that had erupted buccally. An anterior open bite was present with a reverse overjet of 1 mm (Figure 2). Additionally, the lower midline was shifted 3 mm to the right side of the upper midline, which was coincident with the facial midline.

**Figure 3:**
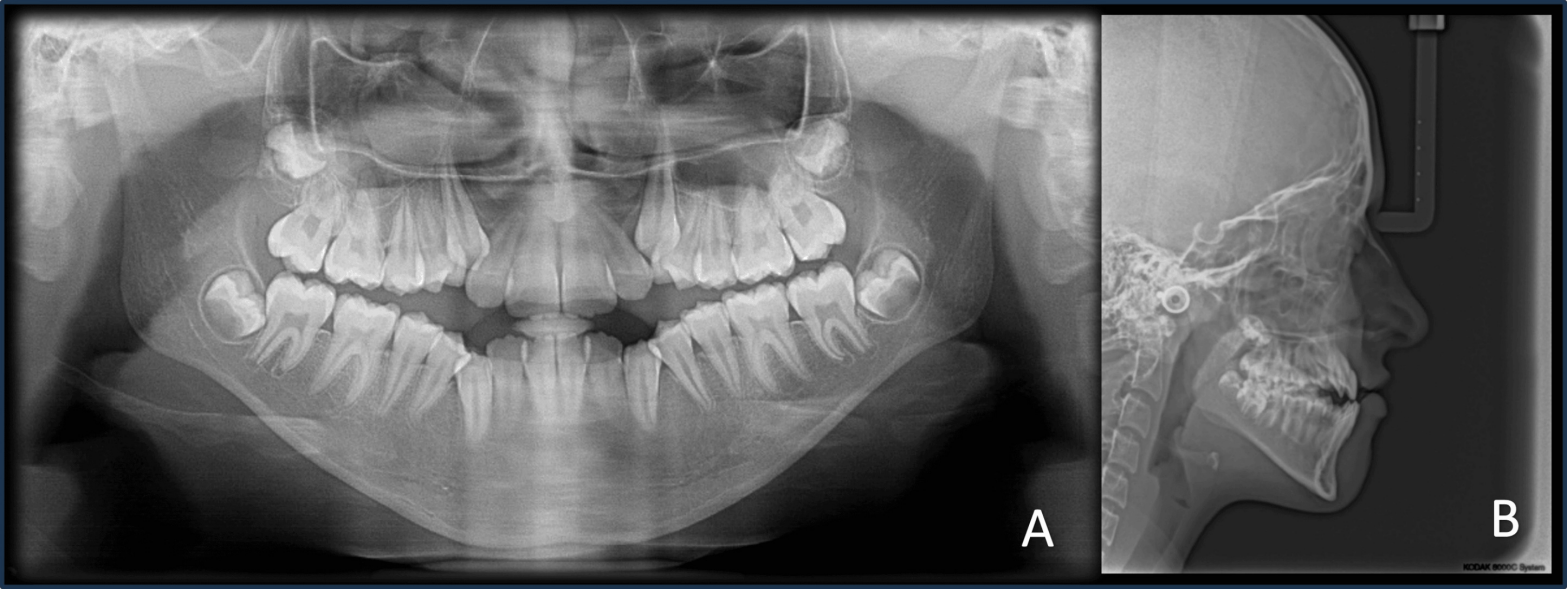
Pre-treatment radiographs A) Panoramic view; B) Lateral cephalogram

The panoramic radiograph (Figure 3A) showed unerupted maxillary right and left canines with completely formed roots. The lateral cephalogram (Figure 3B) revealed Stage 3 of the Cervical Vertebra Maturation Index (CVMI), i.e., transition stage, indicating a remaining pubertal growth of 25% to 65%. There was a skeletal Class III jaw-base relation due to the backwardly placed maxilla (Sella-Nasion to A point (SNA) = 77 degrees) and normally placed mandible (Sella-Nasion to B point (SNB) = 79 degrees), leading to a discrepancy in the jaw-base relationship (A point-Nasion-B point angle (ANB) = -2 degrees). The cephalometric analysis further revealed an effective maxillary length of 73 mm, whereas the effective length of the mandible increased to 110 mm, according to McNamara [8], indicating a large size of the mandible in comparison to the maxilla. The Wits appraisal revealed a discrepancy of -6 mm, and the beta angle was found to be 39 degrees, indicating a Class III malocclusion.

Furthermore, the patient had an increased lower anterior facial height of 67 mm, a decreased Jarabak’s ratio of 54%, and a Frankfort mandibular plane angle (FMA) of 36 degrees, suggesting an underlying vertical growth pattern. The base plane angle of 32 degrees also indicated a hyperdivergent profile. The dental parameters showed upright upper and lower incisors (upper incisor to Nasion-A point (NA) = 25 degrees/4 mm; lower incisor to Nasion-B point (NB) = 22 degrees/4 mm). The incisor mandibular plane angle (IMPA) was 88 degrees, which also indicated an upright positioning of the lower incisors. The soft tissue analysis revealed a decreased nasolabial angle of 88 degrees and a normal relation of the upper and lower lips to the S and E lines (Table 1).

**Table 1:** Findings of cephalometric analysis FH-SN: Frankfort Horizontal to Sella Nasion; SNA: Sella-Nasion-A point angle; SNB: Sella-Nasion-B point angle; SND: Sella-Nasion-D point angle; FMA: Frankfort mandibular plane angle; Wits AO BO: Wits appraisal (A point to B point); U1: Upper incisor; L1: Lower incisor; LAFH: Lower anterior facial height; IMPA: Incisor mandibular plane angle; ANB: A point-Nasion-B point angle; deg: Degrees

Parameters	Pre-treatment	Post-treatment
FH-SN (deg)	7	7
Cant of occlusion	7	8
Maxilla		
SNA (deg)	77	78
N perpendicular to A (mm)	-3	-2
Effective maxillary length (mm)	72	73
Mandible		
SND (deg)	77	76
SNB (deg)	79	78
N perpendicular to Pog (mm)	1	0
Effective mandibular length (mm)	110	109
Maxilla-mandible		
Wits AO BO (mm)	-6	-3
ANB (deg)	-2	0
Beta angle (deg)	39	35
Angle of convexity (deg)	-2	1
Maxillo-mandibular difference (mm)	38	36
Vertical parameters		
FMA (deg)	36	36
SnGoGn (deg)	43	43
Y-axis (deg)	61	61
LAFH (mm)	67	68
Jarabaks ratio	54	54
Saddle angle (deg)	127	127
Articular angle (deg)	140	142
Gonial angle (deg)	138	139
Björk's sum	405	408
Base plane angle (deg)	32	32
Inclination angle (deg)	82	83
Dental parameters - maxillary incisor		
U1-NA (deg/mm)	25/4	26/5
U1-A Pog (mm)	3	4
U1-A vert (mm)	4	4
U1-FH (deg)	110	112
U1-SN (deg)	102	104
Dental parameters - mandibular incisor		
L1-NB (deg/mm)	22/4	20/3
IMPA (deg)	88	85
L1-occlusal plane (deg)	11	9
L1-A Pog (mm)	3	2
Dental parameters - Max-Mand incisor		
Interincisal angle (deg)	139	138
Overjet (mm)	-1	2
Dental parameters - Vertical		
Overbite (mm)	0	2
Curve of Spee (mm)	2	1.5
Soft tissue		
Nasolabial angle (deg)	88	95
Lip strain (mm)	1	0
Upper lip - S line (mm)	-2	-1
Lower lip - S line (mm)	0	-1
Lower lip - E line (mm)	-1	-1

Treatment objectives and planning

The treatment objectives were to correct the skeletal Class III discrepancy, expand the maxillary arch, facilitate the eruption of the maxillary canines, relieve crowding in the mandibular arch, control the vertical growth pattern, achieve ideal overjet and overbite, establish Angle’s Class I molar and canine relation on both sides and create an overall harmonious soft tissue profile.

Since the patient had CVMI Stage 3, there was enough growth remaining for growth modulation therapy. Therefore, maxillary protraction was planned for the patient, using facemask therapy along with rapid maxillary expansion with the Hyrax appliance. Considering the vertical growth pattern of the patient, a bonded Hyrax appliance was chosen, which covered the occlusal surfaces of the posterior teeth with an acrylic plate. Reassessment of the occlusion was planned after achieving maxillary expansion during the first phase of treatment.

Treatment progress

A signed informed consent was obtained from the parents before starting the treatment. A bonded Hyrax appliance was cemented in the maxillary arch for rapid palatal expansion. A hook on each side was incorporated for the attachment of elastics for maxillary protraction. The activation of the appliance was started 24 hours after cementation, following the protocol given by Zimring and Isaacson [9]. The patient was instructed to activate the appliance by turning the screw 90 degrees once in the morning and once in the evening for the next five days. Afterward, only one 90-degree turn was advised for each day until the desired expansion was achieved.

**Figure 4:**
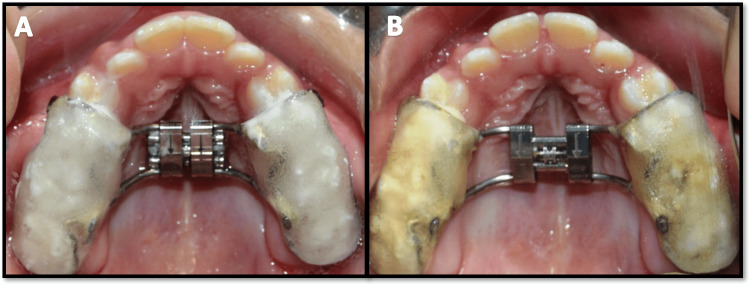
Rapid palatal expansion A) Bonded Hyrax appliance placement; B) Midline diastema observed after 15 days of activation

**Figure 5:**
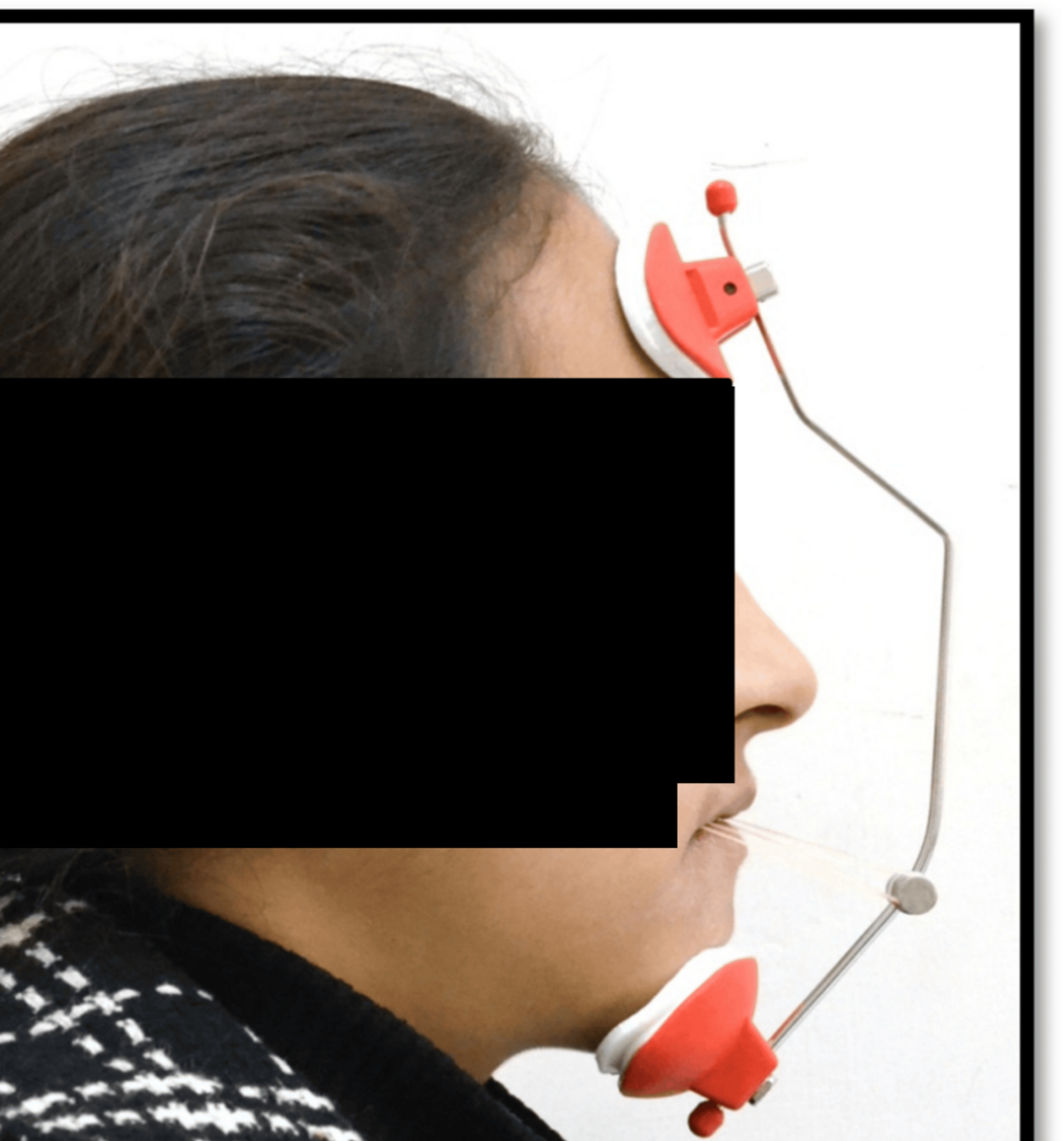
Petit facemask therapy for maxillary protraction

A midline diastema was observed 15 days after starting the expansion, indicating separation of the mid-palatine suture (Figure 4). Maxillary protraction was initiated with a petit facemask, which was adjusted according to the patient’s face to facilitate the attachment of extraoral elastics from the hooks incorporated in the expansion appliance. The elastics were placed at an angle of 30 degrees to the occlusal plane (Figure 5). For the initial seven days, extraoral elastics of size 5/16 inches were placed bilaterally to deliver approximately 200 grams of force on each side. The force levels were later increased to 450 grams per side. The patient was instructed to wear the facemask appliance for at least 12 to 14 hours every day.

**Figure 6:**
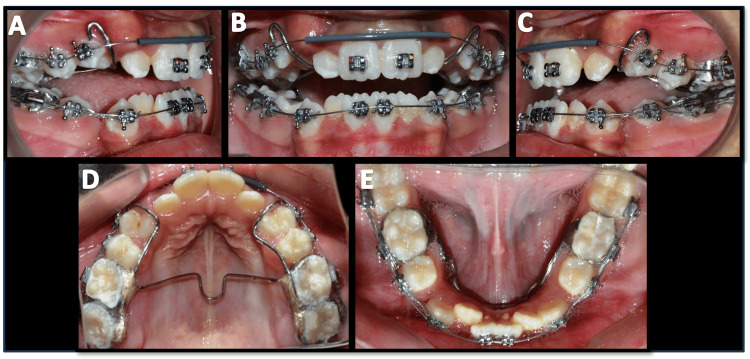
Mid-treatment intra-oral photographs A) Right buccal view; B) Frontal view; C) Left buccal view; D) Maxillary occlusal view; E) Mandibular occlusal view

Treatment overcorrection was carried out, considering the high chances of relapse after palatal expansion, and activation was stopped once the palatal cusps of the maxillary molars and premolars were in contact with the buccal cusps of the mandibular molars and premolars. The bonded Hyrax appliance was left in place for the next three months for retention. After six months of treatment, the expansion appliance was removed, and a trans-palatal arch was placed. It included an extended wire on the palatal side, crossing over the alveolar ridge in the canine region and turning into a hook labially for the attachment of elastics (Figure 6). Considering the severe crowding in the mandibular arch and the space requirement for unerupted canines in the maxillary arch, all four first premolars were extracted, and fixed orthodontic treatment with a 0.22 MBT appliance was initiated. Initially, only the mandibular canines and posterior teeth were bonded, while the incisors were bypassed to upright the distally tipped mandibular canines. A lingual holding arch was installed in the lower arch to augment the maximum anchorage requirement. After uprighting and retraction of the mandibular canines, the incisors were also bonded and included in the main archwire.

The maxillary lateral incisors were not bonded initially to facilitate the eruption of the adjacent canines. Once the maxillary canines had partially erupted, the brackets were placed on the lateral incisors as well. The maxillary canines were brought into alignment using a piggyback 0.14 NiTi archwire, along with a 0.018 Australian stainless steel (SS) main archwire. Space closure was achieved in both arches using sliding mechanics on a rigid 0.019 x 0.025 SS archwire. Once the desired occlusion was achieved, the protraction facemask therapy was stopped, and the fixed MBT appliance was debonded.

Retention was provided by bonded lingual retainers in both the maxillary and mandibular arches. A Begg’s wrap-around retainer was also given to the patient in the maxillary arch to be worn for 24 hours a day for six months. To control mandibular growth, a vertical pull chin cup was provided, and the patient was asked to wear it for at least 12 hours per day until growth was complete. The patient was kept on regular follow-up to assess any future relapse until growth was completed.

Treatment result

**Figure 7:**
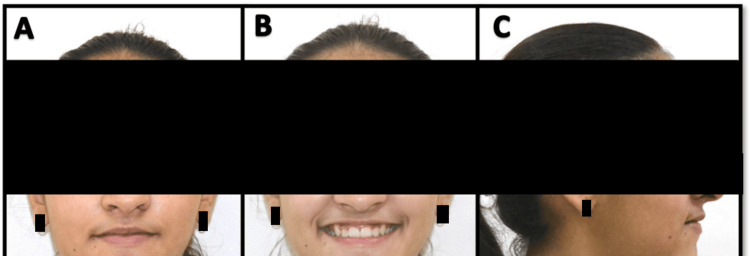
Post-treatment extra-oral photographs A) Frontal at rest; B) Frontal smiling; C) Lateral at rest

**Figure 8:**
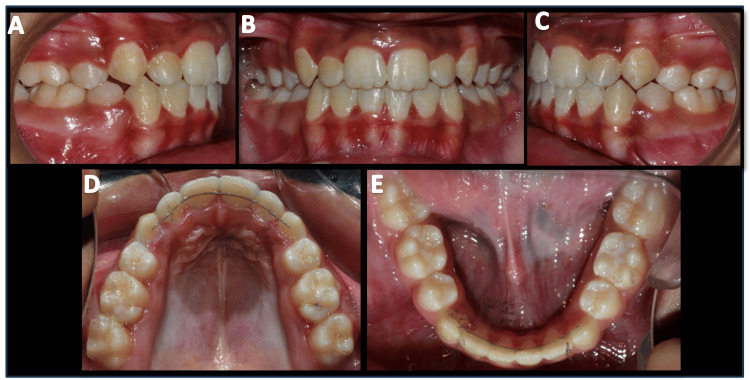
Post-treatment intra-oral photographs A) Right buccal view; B) Frontal view; C) Left buccal view; D) Maxillary occlusal view; E) Mandibular occlusal view

The treatment took 19 months to complete, including the duration of fixed orthodontic treatment after maxillary expansion. At the end of the treatment, an aesthetic and functionally stable occlusion was achieved, and the patient was satisfied. The molars were in a Class I cusp-to-fossa relationship, along with a satisfactory canine relation. An overjet and overbite of 2 mm were also achieved, contributing to a harmonious soft tissue profile. Additionally, the upper and lower midlines matched the facial midline (Figures 7-8).

**Figure 9:**
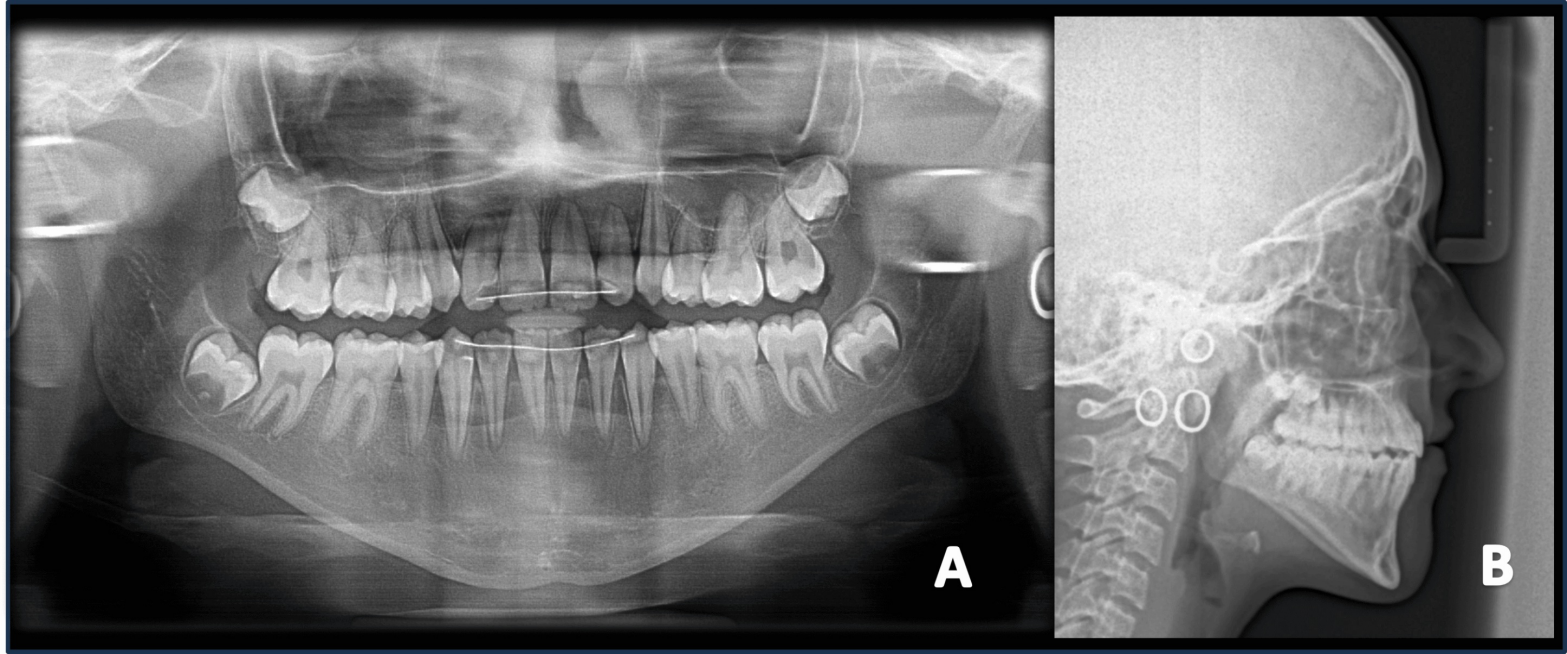
Post-treatment radiographs A) Panoramic view; B) Lateral cephalogram

The post-treatment panoramic view depicted proper root paralleling at the end of the treatment, without any signs of adverse treatment effects (Figure 9). The cephalometric analysis revealed an improvement in the maxillomandibular relationship with facemask therapy, as an improved ANB angle of 0 degrees and a beta angle of 35 degrees were observed. The angle of convexity also improved by 3 degrees. The Wits appraisal revealed a reduced value of -3 mm from the pre-treatment value of -6 mm. There was slight proclination of the upper incisors (upper incisor to NA = 26 degrees/5 mm), whereas the lower incisors were retroclined (lower incisor to NB = 20 degrees/3 mm). The IMPA also reduced to 85 degrees, indicating retroclination of the lower incisors. This was desirable, as upper incisors are slightly proclined and lower incisors are retroclined during camouflage treatment in patients with a Class III skeletal pattern.

**Figure 10:**
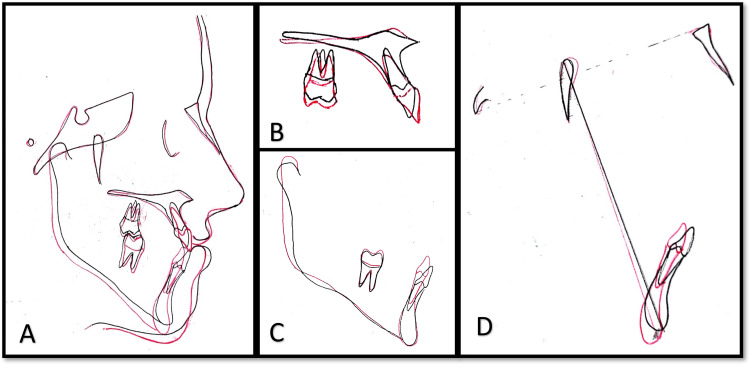
Cephalometric superimpositions A) Overall superimposition; B) Maxillary superimposition; C) Mandibular superimposition; D) Mandible in relation to the cranial base The black color indicates pre-treatment tracing and the red color indicates post-treatment tracing Image credit: Dr. Sana Bint Aziz

The vertical parameters were controlled during the treatment, as indicated by an unchanged FMA of 36 degrees. However, the lower anterior facial height was found to have increased by 1 mm, which could be well appreciated in the superimpositions of the pre-treatment and post-treatment cephalometric tracings. Additionally, the nasolabial angle increased from 88 degrees to 95 degrees, along with a positive lip step (Figure 10).

## Discussion

The treatment goals concerning maxillary expansion and protraction were achieved with orthopedic appliance therapy, along with rapid palatal expansion. The corrective orthodontics enabled the eruption of maxillary canines and bite closure. As the patient was in the pre-pubertal stage, there was an opportunity for growth modulation. Therefore, maxillary protraction with the facemask appliance was carried out to take advantage of the growth potential. It has been reported that the facemask appliance works best during the peak growth period, around 9-11 years of age. The probability of effective maxillary protraction has been found to reduce to zero once sexual maturation is attained [10].

The decision regarding early treatment of developing Class III malocclusion has to be made for the improvement of the skeletal discrepancy. This also allows for future camouflage treatment and reduces the likelihood of orthognathic surgery later on. In the present case report, a petit type of facemask appliance was used for protracting the maxilla and improving its position with the mandible. The elastics were attached from the facemask to intraoral hooks in the maxillary canine region at an angle of 30 degrees to the occlusal plane. This helped prevent undesirable rotation of the palatal plane during maxillary protraction. A bonded type of Hyrax appliance was used for rapid palatal expansion, as the patient had a hyperdivergent profile, and the acrylic blocks over the posterior teeth provided a bite plane effect in this case. Effective expansion, along with improved skeletal discrepancy, was observed with rapid palatal expansion and facemask therapy, as the ANB angle changed from -2 degrees to 0 degrees. This was in agreement with a previous study [11], which observed positive skeletal changes with a 2.6-degree change in the ANB angle and correction of reverse overjet in about 70% of the cases treated with a facemask for maxillary protraction. It was also noted that only 36% of patients who used facemasks required orthognathic surgery later in life, whereas almost 66% of patients who did not use facemasks needed surgery in the future [12].

The present case study observed an increased lower anterior facial height after maxillary protraction. Previous studies [13,14] have encountered problems in controlling the lower anterior facial height and vertical growth pattern with both dental and skeletal anchorage during maxillary protraction. The treatment mechanics were used cautiously to prevent any further worsening of the vertical growth pattern, including a posterior bite plane during expansion to prevent the eruption of posterior teeth and control the lower anterior facial height. In addition, the extraction of premolars was performed for effective bite closure and relieving anterior crowding. However, the presence of a severe vertical growth pattern necessitates consideration of a genioplasty procedure in the future for correction of the prominent chin and increased vertical dimension.

The presence of an anterior open bite further complicated an already complex case due to the higher chances of relapse, thereby requiring long-term retention plans. Open bite closure was achieved with corrective orthodontics after the extraction of all first premolars. A previous case report [15] demonstrated a multidisciplinary approach using the Multiloop Edgewise Archwire (MEAW) technique, along with temporary anchorage devices, for effective closure of an anterior open bite in an adult patient. The stress distribution was found to be more uniform with the use of the MEAW technique compared to straight wire, along with more control of the vertical dimension.

Since the patient was still growing, a vertical pull chin cup was given to control mandibular growth. It was recommended to be worn until the completion of facial growth. However, its use in hyperdivergent patients is controversial, as it causes downward and backward rotation of the mandible [16]. A recent study [17], based on low-dose computed tomography, suggested that chin cup therapy did not affect the dimensions of the mandible. Its major action could, however, be seen in the condyle and the temporomandibular joint [17].

Also, at the end of the orthodontic treatment, it was noted that the patient had a gummy smile. Therefore, a gingivoplasty was suggested as a corrective measure for better display of the maxillary anterior teeth; however, the patient chose not to undergo the treatment at that time. Gingivoplasty after orthodontic treatment has proved to be a minimally invasive and cost-effective measure for the correction of more than 4 mm of gingival display on smile [18].

With the continuous technological transformation in diagnostic tools and newer innovations in orthodontic appliances, treatment options have become more effective as well as patient-friendly, such as the use of temporary skeletal anchorage devices and digital planning. An interdisciplinary approach that combines orthodontics and surgery with advanced technology would improve both treatment outcomes and duration in patients with complex malocclusions.

## Conclusions

The case report describes the impact of rapid palatal expansion and facemask therapy on a pre-pubertal female patient with a skeletal Class III malocclusion, vertical growth pattern, unerupted maxillary canines, and an anterior open bite. An aesthetically improved functional occlusion was obtained at the end of the treatment. However, further surgery may be required in the future due to a prominent chin and an increased vertical dimension.

When addressing skeletal Class III patients with a high mandibular plane angle, early intervention should be approached with caution, taking into account both positive and negative factors. It is important to recognize that some patients may still require orthognathic surgery in the future. Nevertheless, the primary objective of early intervention in skeletal Class III cases is to correct the skeletal imbalance, eliminate any discrepancies in centric relation and centric occlusion, and reduce the likelihood of future orthognathic surgery.
